# Integrative Pathway Analysis of Metabolic Signature in Bladder Cancer: A Linkage to The Cancer Genome Atlas Project and Prediction of Survival

**DOI:** 10.1016/j.juro.2016.01.039

**Published:** 2016-06

**Authors:** Friedrich-Carl von Rundstedt, Kimal Rajapakshe, Jing Ma, James M. Arnold, Jie Gohlke, Vasanta Putluri, Rashmi Krishnapuram, D. Badrajee Piyarathna, Yair Lotan, Daniel Gödde, Stephan Roth, Stephan Störkel, Jonathan M. Levitt, George Michailidis, Arun Sreekumar, Seth P. Lerner, Cristian Coarfa, Nagireddy Putluri

**Affiliations:** a Scott Department of Urology, Baylor College of Medicine, Houston, Texas; b Department of Molecular and Cell Biology, Alkek Center for Molecular Discovery, Baylor College of Medicine, Houston, Texas; c Verna and Marrs McLean Department of Biochemistry, Baylor College of Medicine, Houston, Texas; d Advanced Technology Core, Baylor College of Medicine, Houston, Texas; e Department of Pathology and Immunology, Baylor College of Medicine, Houston, Texas; f Department of Urology, University of Texas Southwestern Medical Center, Dallas, Texas; g Department of Urology, Witten-Herdecke University, Wuppertal, Germany; h Department of Pathology Helios Klinikum, Witten-Herdecke University, Wuppertal, Germany; i Department of Statistics, University of Michigan, Ann Arbor, Michigan

**Keywords:** urinary bladder neoplasms, urothelium, metabolomics, mass spectrometry, metabolic networks and pathways, BCa, bladder cancer

## Abstract

**Purpose:**

We used targeted mass spectrometry to study the metabolic fingerprint of urothelial cancer and determine whether the biochemical pathway analysis gene signature would have a predictive value in independent cohorts of patients with bladder cancer.

**Materials and Methods:**

Pathologically evaluated, bladder derived tissues, including benign adjacent tissue from 14 patients and bladder cancer from 46, were analyzed by liquid chromatography based targeted mass spectrometry. Differential metabolites associated with tumor samples in comparison to benign tissue were identified by adjusting the p values for multiple testing at a false discovery rate threshold of 15%. Enrichment of pathways and processes associated with the metabolic signature were determined using the GO (Gene Ontology) Database and MSigDB (Molecular Signature Database). Integration of metabolite alterations with transcriptome data from TCGA (The Cancer Genome Atlas) was done to identify the molecular signature of 30 metabolic genes. Available outcome data from TCGA portal were used to determine the association with survival.

**Results:**

We identified 145 metabolites, of which analysis revealed 31 differential metabolites when comparing benign and tumor tissue samples. Using the KEGG (Kyoto Encyclopedia of Genes and Genomes) Database we identified a total of 174 genes that correlated with the altered metabolic pathways involved. By integrating these genes with the transcriptomic data from the corresponding TCGA data set we identified a metabolic signature consisting of 30 genes. The signature was significant in its prediction of survival in 95 patients with a low signature score vs 282 with a high signature score (p = 0.0458).

**Conclusions:**

Targeted mass spectrometry of bladder cancer is highly sensitive for detecting metabolic alterations. Applying transcriptome data allows for integration into larger data sets and identification of relevant metabolic pathways in bladder cancer progression.

Urothelial BCa is the second most prevalent urological malignancy and the fourth most common cause of cancer in men in the United States.^1^ Because most patients present with nonmuscle invasive BCa at the time of diagnosis, this cancer can be managed by bladder sparing approaches. Conversely most patients who present with gross hematuria will have muscle invasive disease. The biological processes that are associated with tumor progression are poorly understood.

While radical cystectomy offers excellent survival rates for organ confined disease, the outcome is inferior in patients with advanced disease as well as those who undergo delayed cystectomy for nonmuscle invasive BCa.^2^ A significant limitation in the management of advanced disease is the inability to predict the patients who are most likely to respond to systemic chemotherapy. A wide spectrum of urinary and molecular markers has been evaluated but none have been shown to predict the natural history of BCa in the clinical setting.^3^ Physicians currently rely predominantly on histological grade and primary tumor stage from initial bladder tumor resection as well as cross-sectional imaging to assess the risk of disease progression in individual patients.

Unlike other tumors BCa is closely linked to environmental carcinogens, including tobacco, polyaromatic hydrocarbons, aniline, benzidine, naphthylamine and multiple other compounds.^4^ Our group has previously performed unbiased metabolic profiling of bladder derived tissues and was able to report the accumulation of aromatic compounds and xenobiotic compounds in tumors.^5^ In this context elevated levels of SAM (S-adenosyl methionine) were indicative of increased methylation potential in these tumors. Notably the latter contributes to the decreased expression of enzymes in the cytochrome driven phase I metabolic pathway, which could potentially lead to alterations in precarcinogen metabolism in bladder tissues.

A recently published, comprehensive molecular analysis of urothelial BCa from TCGA (http://cancergenome.nih.gov/) has provided us with novel insights into molecular subgroups and potential targets for targeted therapies.^6^ We have asked the question of whether we could define a metabolic gene signature associated with progression and survival by integrating metabolomic pathway analysis based on a validated targeted mass spectrometry platform with TCGA transcriptome profiles. Our report provides insight into metabolic pathways that are potentially associated with tumorigenesis and tumor progression.

## Patients and Methods

### Bladder Tumor Metabolomic Profiling

A total of 60 pathologically verified tissues, including benign adjacent tissue from 14 patients and BCa from 46 (table 1), were obtained from the tumor banks of the participating institutions (Baylor College of Medicine, University of Texas Southwestern Medical Center and University of Witten-Herdecke). All samples were de-identified and included in study under an institutional review board approved protocol (H-35808).

**Table 1:** Clinical characteristics of 60 patients

	No. Pts (%)
Pathological stage:	
Ta	1 (2)
T1	1 (2)
T2	12 (19)
T3	23 (37)
T4	9 (14)
Normal	14 (22)
Grade:∗	
High	44 (96)
Missing	2 (4)
Lymph node metastasis:	
Present	21 (45)
Absent	25 (53)
Neoadjuvant chemotherapy:	
Present	6 (13)
Absent	40 (85)

∗ No low grade BCa.

Flash frozen, macrodissected tumor samples as well as adjacent noncancerous tissues were reviewed by a genitourinary pathologist. Of 46 tumor samples 44 were muscle invasive bladder tumors and 21 patients were found to have lymph node metastasis at the time of tissue collection. Tissue was retrieved at transurethral tumor resection or cystectomy. All patients ultimately underwent radical cystectomy and pathological tumor stage was available for all. Six of the 46 patients received neoadjuvant chemotherapy.

**Figure 1:**
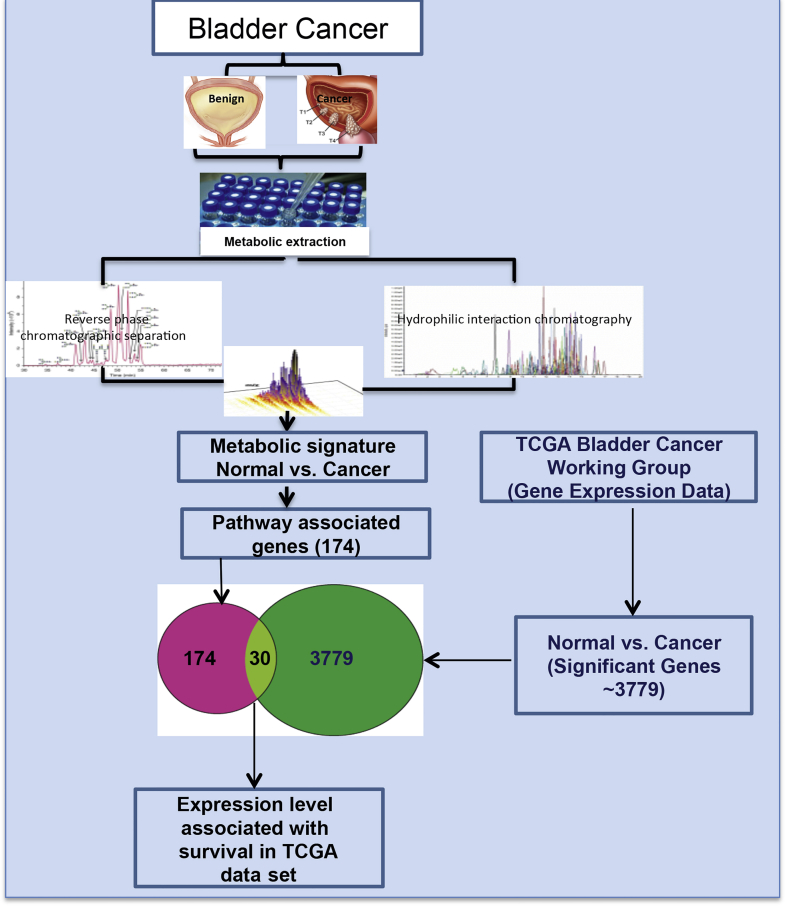
Overview of strategy used to profile and characterize BCa metabolome

Tissues were analyzed by liquid chromatography-mass spectrometry. The metabolome was separated using reverse phase and aqueous normal phase chromatography, and examined by mass spectrometry using positive and negative electrospray ionization. The mass spectrometer was used to examine 145 metabolites by single reaction monitoring. Figure 1 shows a schematic overview of the metabolomic profiling and the associated data analysis strategy used in this study. The chromatographic separation of metabolites and analysis by mass spectrometry were monitored for drifts using internal standards and biological replicates of pooled extract of mouse liver and were found to be highly reproducible.

### Liquid Chromatography/Mass Spectrometry

Samples were injected and analyzed using the 6490 Triple Quadrupole mass spectrometer coupled to a high performance liquid chromatography system (Agilent®) via single reaction monitoring of 145 endogenous water soluble metabolites for steady-state analyses of samples. The 145 compounds monitored were chosen due to involvement in central pathways important in a number of malignancies. Source parameters were gas temperature 250C, gas flow 14 L per minute, nebulizer 20 psi, sheath gas temperature 350C, sheath gas flow 12 L per minute, capillary 3,000 V positive and 3,000 V negative, and nozzle voltage 1,500 V positive and 1,500 V negative.

Approximately 8 to 11 data points were acquired per detected metabolite. Samples were delivered to the mass spectrometer via normal phase chromatography using a 4.6 mm inner diameter × 10 cm XBridge™ HILIC Amide Column or a Luna® 3 μ NH2 100 Å Column at 300 μl per minute. Gradients were run starting from 85% buffer B (high performance liquid chromatography grade acetonitrile or 0.1% formic acid in acetonitrile) to 35% B from 0 to 3.5 minutes and from 35% B to 2% B from 3.5 to 11.5 minutes with 2% B held from 11.5 to 16.5 minutes, 2% B to 85% B held from 16.5 to 17.5 minutes and 85% B held for 7 minutes to re-equilibrate the column. The peak area of each metabolite was integrated using MassHunter Workstation Software Quantitative Analysis, version B.06.00 (Agilent).

### Statistical Analysis

Metabolite levels were normalized relative to the median level of spiked internal standards (zeatin and tryptophan 15 N2) separately for all 4 methods used. Data were log2-transformed and median centered. For every metabolite in the normalized data set the 2-sample t-test was applied to compare expression levels in BCa and adjacent benign tissues. Differential metabolites were identified after adjusting p values for multiple testing at a false discovery rate threshold of 15%. Hierarchical clustering of differential metabolites was generated using the R statistical system (https://www.r-project.org/).

### Integration of Metabolomic and Transcriptomic Data

We integrated the common metabolites with urothelial BCa transcriptome data published by TCGA. Metabolites were converted to KEGG Database (http://www.genome.jp/kegg/kegg1.html) enzyme/gene IDs using an in-house database. We determined the enrichment of pathways and biological processes in this signature using the collections compiled by the GO Database (http://geneontology.org/page/go-database) and MSigDB (http://software.broadinstitute.org/gsea/msigdb/). We performed overrepresentation analysis of pathways based on the hypergeometric distribution (p <0.05) of the biochemical genes.

Next we obtained the transcriptome fingerprint between matched normal and tumor tissues in TCGA urothelial BCa data set (fold change exceeding twofold, p <0.05). We generated an integrated metabolomics/transcriptomics gene signature by intersecting the mentioned gene sets. A total of 30 genes found to be differential between BCa and matched adjacent benign tissues in TCGA were also associated with metabolites significantly altered between tumor and adjacent matched benign tissues. This set of 30 genes and the associated fold changes in TCGA data were termed the integrated metabolomic/transcriptomic gene signature (table 2).

**Table 2:** 

Gene	Log2_FC(tumor/benign)
XDH	4.28
TDO2	3.73
GAD1	2.88
CHIT1	2.86
DNMT3B	2.54
PYCR1	2.53
KMO	1.83
TYMP	1.52
PYCRL	1.45
SUV420H2	1.2
B4GALT3	1.14
LYPLA2	1.08
PLA2G15	1.08
DNMT1	1
NNMT	−1.01
BST1	−1.05
TARSL2	−1.1
SETD7	−1.12
GPD1	−1.13
GPD1L	−1.19
EXTL1	−1.32
GATM	−1.33
GAMT	−1.43
ALDH7A1	−1.64
DPYD	−1.66
ALDH1B1	−1.76
PLA2G4A	−1.77
PIPOX	−1.96
ALDH2	−2.37
INMT	−2.61

### Analysis of Integrated Tumor Progression Gene Signature Prognosis Power

The significant association with survival of an integrated metabolomic/transcriptomic gene signature was evaluated in a gene expression data set corresponding to the urothelial BCa patient cohort from TCGA working group, for which clinical outcomes have been reported. For each gene in the signature and for each specimen we calculated the z-score for expression in the cohort as described previously.^7^ We then calculated the sum z-score for each specimen. Specifically the z-scores of genes repressed in the integrated signature were subtracted from the z-scores of genes induced in the integrated signature, resulting in a corresponding signature activity score for each specimen. In each data set specimens were ranked according to the integrated signature activity score and the association with survival was evaluated by the log-rank test between the bottom 25% and the top 75% of the patients. Survival significance was assessed by the survival package in R.

### Generation of Focused Integrated Gene Signature Associated with Prognosis in Multiple Cohorts

We analyzed the association of the integrated 30-gene signature with survival in additional BCa cohorts, including those reported by Kim et al (GSE13507)^8^ and Lindgren et al (GSE32548).^9^ Using the exploratory approach of partitioning samples based on multiple thresholds (eg comparing the top 33% to the bottom 33%, the top 33% to the bottom 67%, etc) described by Budczies et al^10^ we determined that the 30-gene signature was associated with progression in all 3 cohorts, although at different thresholds.

To generate a robust signature with potential clinical application we evaluated whether a subset of the 30 genes could be associated with prognosis using the same breakdown in all 3 cohorts. We first selected 13 genes associated with survival in the same direction in all 3 data sets regardless of the sample breakdown. By applying combinatorial analysis we next determined a subset signature associated with survival at the bottom 25%-top 75% sample breakdown. This consisted of the genes CHIT1, DNMT1, GPD1, PLA2G4A, TARSL2 and SETD7.

## Results

### Bladder Cancer Metabolomic Profiling

**Figure 2:**
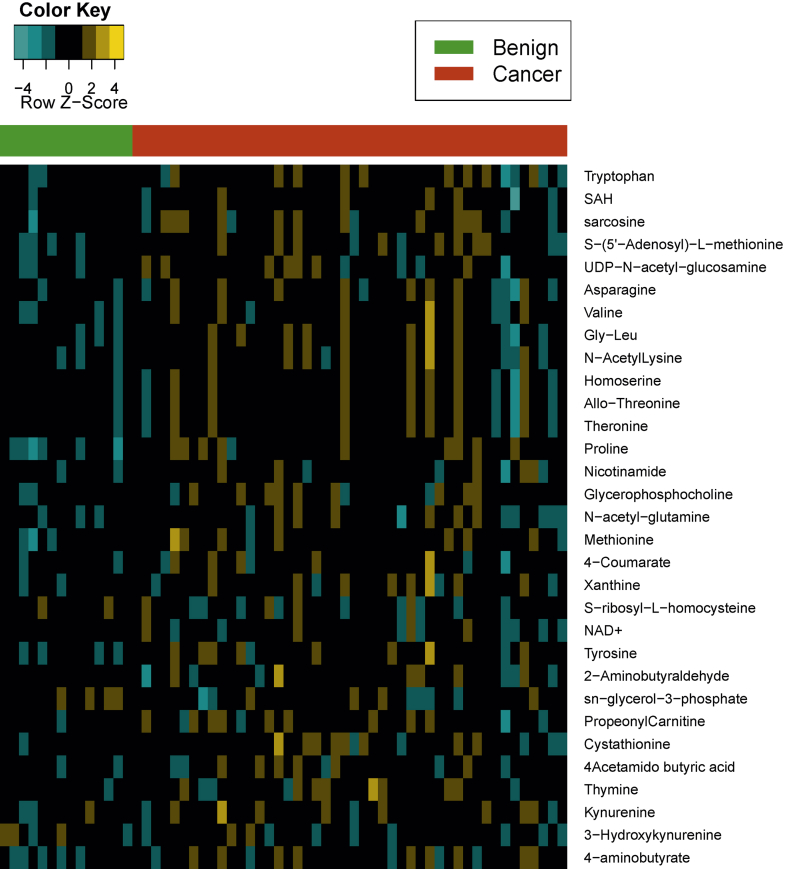
Metabolomic alteration in BCa and heat map shows 31 named differential metabolites in urothelial cancer relative to benign samples. Columns represent individual tissue samples. Rows represent distinct metabolites. Yellows indicate metabolite elevation. Greens indicate metabolite decrease relative to average level (*Color Key*).

We analyzed 60 bladder derived tissues, including 14 benign adjacent tissues and 46 BCa tissues, for metabolomic profiles using liquid chromatography-mass spectrometry. A total of 145 metabolites were measured on the platform across the 60 tissue samples. There was no difference in metabolite levels in the tumor cohort with regard to tumor stage or presence of metastatic disease. However 31 of these metabolites were differentially altered between tumor and benign samples (fig. 2).

From these data we were able to categorize all differential metabolites into various subgroups based on function and/or class. These classifications included metabolites associated with tryptophan metabolism (tryptophan, kynurenine and 3-hydroxykynurenine), the methylation pathway (SAH, SAM, S-ribosyl-homocysteine, methionine and cystathionine), the glycine, serine and threonine pathway (sarcosine, asparagine, homoserine and threonine), the nicotinate and nicotinamide pathway (NAD and nicotinamide), nucleotides/purine metabolism (xanthine and thymine), acetyl amino acids (acetyl lysine, UDP-acetyl-glucosamine, acetyl-glutamine and acetyl butyric acid) and other metabolites (valine, glycine-leucine, proline, 4-coumarate, tyrosine and 2-aminobutyraldehyde, propionyl carnitine, and 4-aminobutyrate).

### Robust Metabolic Changes between Adjacent Normal and Tumor Tissues

**Figure 3:**
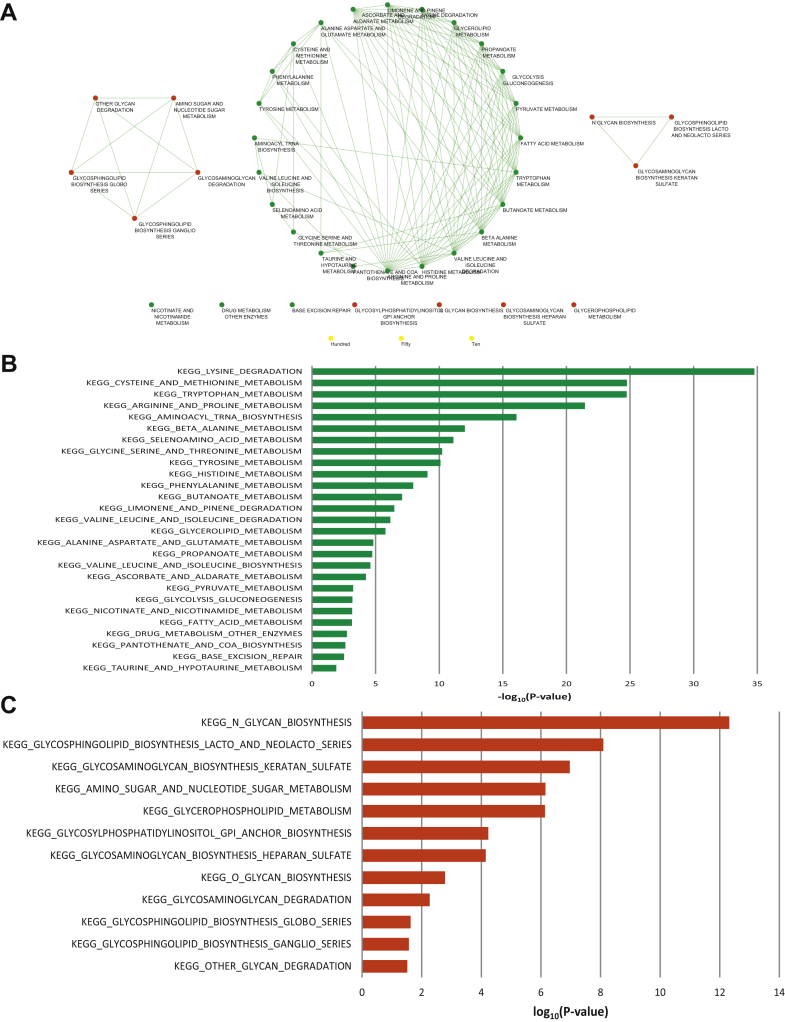
*A*, enriched pathways and processes in metabolomic gene signature (hypergeometric distribution p <0.05). Differential set of metabolites was mapped to corresponding genes, which were used to enrich for biochemical pathways. Red indicates pathways associated with current metabolic signature. Green indicates pathways associated with previous metabolic signature.^17^*B*, pathways common to current and previous^17^ metabolic signatures. *C*, novel pathways from current metabolic signature.

To further obtain insights into altered biochemical pathways we mapped the differential set of metabolites to the corresponding genes, which were then used to enrich for biochemical pathways. Pathway enrichment analysis nominated 39 significantly enriched biochemical pathways (p <0.05). Of these pathways 24 were highly interconnected and related to the synthesis of amino acids, namely lysine, cysteine, methionine, arginine, proline, tryptophan and alanine as well as glycolysis, lipid metabolism and fatty acids (fig. 3). Pathway network was visualized with Cytoscape software (http://www.cytoscape.org/).

Next we used TCGA to cull the list of differential genes between urothelial carcinoma and benign tissue. A total of 3,779 genes were found to be differential (fold change exceeding twofold, p <0.05). When overlapped with the gene list corresponding to the differential metabolites described, 30 genes were obtained (table 2). These genes were significantly altered between urothelial carcinoma and benign tissue in TCGA, and they were also associated with metabolites significantly altered between benign and BCa tissues. This core set of 30 genes was examined for its prognostic power in TCGA data set.

**Figure 4:**
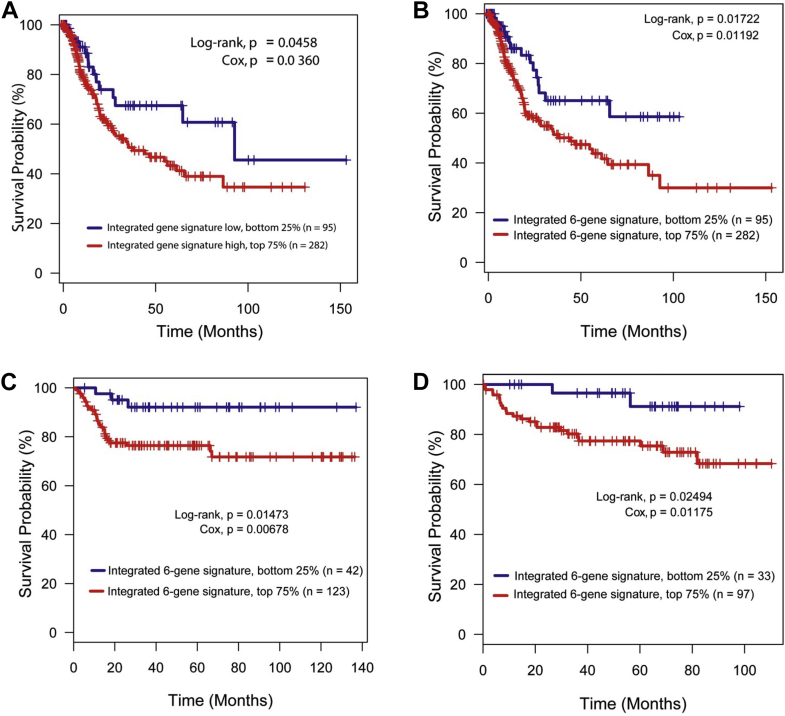
Survival analyses. *A*, in TCGA-BLCA (Bladder Urothelial Carcinoma) cohort for integrated metabolomics/transcriptomics signature, which was significantly associated with worse prognosis in this cohort. *B* to *D*, in BCa cohorts of integrated 6-gene signature consisting of CHIT1, DNMT1, GPD1, PLA2G4A, TARSL2 and SETD7, which was significantly associated with worse prognosis in all 3 cohorts. *B*, TCGA. *C*, Kim et al (GSE13507).^8^*D*, Lindgren et al (GSE32548).^9^

This integrated metabolomic/transcriptomic gene signature was significantly associated with survival in TCGA cohort of patients with urothelial cancer (log-rank test p = 0.0458, Cox proportional hazards p = 0.0360, fig. 4). Patients with a higher activity score for the integrated signature showed a worse prognosis. Using combinatorial analysis we identified a subset 6-gene signature associated with survival in additional cohorts, namely those of Kim^8^ and Lindgren^9^ et al, consisting of the genes CHIT1, DNMT1, GPD1, PLA2G4A, TARSL2 and SETD7 (fig. 4, *C* and *D*).

## Discussion

We report mass spectrometry based, metabolic pathway analysis of urothelial cancer of the bladder. We were able to identify commonly altered biochemical pathways and determine a metabolite derived gene signature that we found was predictive of greater than 10-year survival in TCGA data set. By integrating metabolomic pathway analysis based on a validated targeted mass spectrometry platform with TCGA transcriptome profiles we were able to define a metabolic gene signature associated with progression and survival.^11^ This allows for evaluation of the biological role as well as the clinical relevance of the signature.

The idea of coupling data from different aspects of the same biological system, a term known as integrative analysis, is not new.^12^, ^13^, ^14^, ^15^ In several recent studies this concept was applied to identify gene function or gene-to-metabolite networks but to our knowledge the current data set represents a novel approach to BCa metabolomics.

The scope of our targeted mass spectrometry based analysis involved 145 metabolites, including amino acids, amino sugars, nucleotides, organic acids and fatty acids. A total of 31 metabolites were differentially enriched when comparing benign bladder and bladder tumor samples. Enrichment analysis highlighted multiple biological processes in the enrichment grid with an emphasis on amino acid metabolism, nucleotides, lipids and glycolysis (fig. 4). This is coherent with the metabolic requirements for cell proliferation proposed by Vander Heiden et al.^16^

The Warburg effect is a phenomenon in cancer cells in which they rapidly metabolize glucose to lactate using cytosolic aerobic glycolysis rather than the more efficient generation of adenosine triphosphate through mitochondrial oxidative phosphorylation. While we observed pathway alterations associated with the Warburg effect (glycolysis and pyruvate metabolism), our analysis did not show specific metabolite changes, which are better evaluated by flux analysis. On integrated pathway analysis we found a significant overlap with a previously reported metabolic signature (supplementary figure, http://jurology.com/),^17^ which is supportive of the biological importance.^5^ This is in agreement with reports indicating alterations in amino acid levels and the potential association with tumor development.

Up to 70% of dry cell weight consists of protein, which directly correlates with a demand for protein synthesis. While essential amino acids cannot be synthesized in the cell, the flux profile of the amino acids might be an indicator of protein synthesis dynamics in cancer cells. A recent study revealed a relationship between the amino acid exchange rate and cancer cell proliferation in cell line models.^18^ Metabolomics may be helpful to identify patterns in amino acid metabolism that can be modified by targeted drugs. A valuable example is the effect of mTOR inhibitors on protein synthesis in cancer cells.^19^

A metabolomics approach captures the actual real-time metabolism but single metabolites may not be reflective of a pattern simply because the metabolic state undergoes constant change. On the contrary focusing on pathways rather than on single metabolites appears to be more reflective of important biological processes. The integration of pathway associated genes with the transcriptome of large data sets provides an opportunity to validate the prognostic value of gene signatures. TCGA data set is an excellent resource as it has set high standards with regard to sample collection, sequencing technology and platform analysis.

When applying the 30 metabolic gene signature to TCGA cohort, we were able to observe a significant difference in survival in patients with an enrichment of metabolic genes compared to those without such enrichment. This suggests that up-regulation of these genes may be associated with tumor progression and the pathway alterations from our analysis may be of clinical relevance.

Specifically using the MSigDB platform we determined significant enrichment for multiple metabolic pathways and processes, namely arginine and proline metabolism (p = 4 × 10^–11^, q = 6.93 × 10^–12^), tryptophan metabolism (p = 1.72 × 10^–13^, q = 1.45 × 10^–10^), lysine degradation (p = 3.15 × 10^–13^, q = 1.77 × 10^–10^), β alanine metabolism (p = 2.18 × 10^–12^, q = 9.19 × 10^–10^) and glycerophospholipid metabolism (p = 1.6 × 10^–9^, q = 5.38 × 10^–7^). Notably ALDH7A1, ALDH2 and ALDH1B1 are present in the top 4 enriched pathways. Genes in the aldehyde dehydrogenase family have been previously reported as associated with shorter survival in BCa.^20^ High expression of GAMT (guanidinoacetate N-methyltransferase), which is present in the arginine and proline metabolism pathway, has been associated with better prognosis of BCa. Expression of TDO2 (tryptophan 2,3-dioxygenase) has been reported as a potential target in immunotherapy of multiple cancers, including BCa.^21^ Lastly the increase in glycerophospholipids was observed in canine models of BCa.^22^

We next characterized the focused 6-gene signature and observed significant enrichment for glycerophospholipid metabolism (p = 4.14e–5, q = 2.64e–2) and for glycerophospholipid biosynthesis (p = 4.7 × 10^–5^, q = 2.64 × 10^–2^). They were driven by genes such as PLA2G4A (phospholipase A2, group IVA) and GPD1 (glycerol-3-phosphate dehydrogenase 1). As corroborated in prior studies in animal models of BCa this result points to the potential importance of modulating glycerophospholipids as a BCa therapeutic modality.

Our study has important limitations. Our analysis was performed on a single platform with a limited number of patients. A metabolite derived gene expression signature should ideally be validated in the same data set using gene expression data as well as in independent data sets with long-term clinical followup data.

## Conclusions

Targeted mass spectrometry of BCa is highly sensitive for detecting metabolic alterations. Pathway analysis allows for the integration of metabolomic and transcriptomic profiling to enhance data set size and applicability to biological processes. We have defined an integrated metabolic/transcriptomic pathway signature associated with BCa progression and report its potential value to predict the clinical outcome.

## References

[1] (2014). Cancer Facts and Figures 2014.

[2] Stein J.P., Lieskovsky G., Cote R. (2001). Radical cystectomy in the treatment of invasive bladder cancer: long-term results in 1,054 patients. J Clin Oncol.

[3] Xylinas E., Klute L.A., Lotan Y. (2014). Blood- and tissue-based biomarkers for prediction of outcomes in urothelial carcinoma of the bladder. Urol Oncol.

[4] Kiriluk K.J., Prasad S.M., Patel A.R. (2012). Bladder cancer risk from occupational and environmental exposures. Urol Oncol.

[5] Putluri N., Shojaie A., Vasu V.T. (2011). Metabolomic profiling reveals a role for androgen in activating amino acid metabolism and methylation in prostate cancer cells. PLoS One.

[6] Cancer Genome Atlas Research Network (2014). Comprehensive molecular characterization of urothelial bladder carcinoma. Nature.

[7] He B., Lanz R.B., Fiskus W. (2014). GATA2 facilitates steroid receptor coactivator recruitment to the androgen receptor complex. Proc Natl Acad Sci U S A.

[8] Kim W.J., Kim E.J., Kim S.K. (2010). Predictive value of progression-related gene classifier in primary non-muscle invasive bladder cancer. Mol Cancer.

[9] Lindgren D., Sjödahl G., Lauss M. (2012). Integrated genomic and gene expression profiling identifies two major genomic circuits in urothelial carcinoma. PLoS One.

[10] Budczies J., Klauschen F., Sinn B.V. (2012). Cutoff Finder: a comprehensive and straightforward Web application enabling rapid biomarker cutoff optimization. PLoS One.

[11] Putluri N., Maity S., Creighton C.J. (2014). Pathway-centric integrative analysis identifies RRM2 as a prognostic marker in breast cancer associated with poor survival and tamoxifen resistance. Neoplasia.

[12] Meierhofer D., Weidner C., Sauer S. (2015). Correction to “integrative analysis of transcriptomics, proteomics, and metabolomics data of white adipose and liver tissue of high-fat diet and rosiglitazone-treated insulin-resistant mice identified pathway alterations and molecular hubs.”. J Proteome Res.

[13] Meierhofer D., Weidner C., Sauer S. (2014). Integrative analysis of transcriptomics, proteomics, and metabolomics data of white adipose and liver tissue of high-fat diet and rosiglitazone-treated insulin-resistant mice identified pathway alterations and molecular hubs. J Proteome Res.

[14] Brink-Jensen K., Bak S., Jørgensen K. (2013). Integrative analysis of metabolomics and transcriptomics data: a unified model framework to identify underlying system pathways. PLoS One.

[15] Stanberry L., Mias G.I., Haynes W. (2013). Integrative analysis of longitudinal metabolomics data from a personal multi-omics profile. Metabolites.

[16] Vander Heiden M.G., Cantley L.C., Thompson C.B. (2009). Understanding the Warburg effect: the metabolic requirements of cell proliferation. Science.

[17] Putluri N., Shojaie A., Vasu V.T. (2011). Metabolomic profiling reveals potential markers and bioprocesses altered in bladder cancer progression. Cancer Res.

[18] Dolfi S.C., Chan L.L., Qiu J. (2013). The metabolic demands of cancer cells are coupled to their size and protein synthesis rates. Cancer Metab.

[19] Duvel K., Yecies J.L., Menon S. (2010). Activation of a metabolic gene regulatory network downstream of mTOR complex 1. Mol Cell.

[20] Su Y., Qiu Q., Zhang X. (2010). Aldehyde dehydrogenase 1 A1-positive cell population is enriched in tumor-initiating cells and associated with progression of bladder cancer. Cancer Epidemiol Biomarkers Prev.

[21] Pilotte L., Larrieu P., Stroobant V. (2012). Reversal of tumoral immune resistance by inhibition of tryptophan 2,3-dioxygenase. Proc Natl Acad Sci U S A.

[22] Dill A.L., Ifa D.R., Manicke N.E. (2009). Lipid profiles of canine invasive transitional cell carcinoma of the urinary bladder and adjacent normal tissue by desorption electrospray ionization imaging mass spectrometry. Anal Chem.

