# Supplementary material for: Integrative Pathway Analysis of Metabolic Signature in Bladder Cancer: A Linkage to The Cancer Genome Atlas Project and Prediction of Survival
Source: J Urol. 2016 Jun;195(6):1911–9. doi: 10.1016/j.juro.2016.01.039 (PMC4861129; doi:10.1016/j.juro.2016.01.039)
Supplement: Legend for Supplementary Figure [file mmc1.pdf]

Supplementary Figure. KEGG : Metabolic pathways.

A) Red line indicates the pathways associated with current metabolic signature.

B) Blue line indicates the pathways associated with metabolic signatures from the paper: Putluti, et.al; Cancer Res. 2011 Dec 15;71(24):7376-86 .

C) The green line indicates the pathways associated with gene signature from TCGA-BLCA.

D) Purple line indicates the common pathways from A & B. E)Brown line indicates the common pathways from A & C. E)Cyan indicates the common pathways from B & C. F)Black line indicates pathways common to all.
