# Supplementary figures and images for: Integrative Pathway Analysis of Metabolic Signature in Bladder Cancer: A Linkage to The Cancer Genome Atlas Project and Prediction of Survival
Source: J Urol. 2016 Jun;195(6):1911–9. doi: 10.1016/j.juro.2016.01.039 (PMC4861129; doi:10.1016/j.juro.2016.01.039)

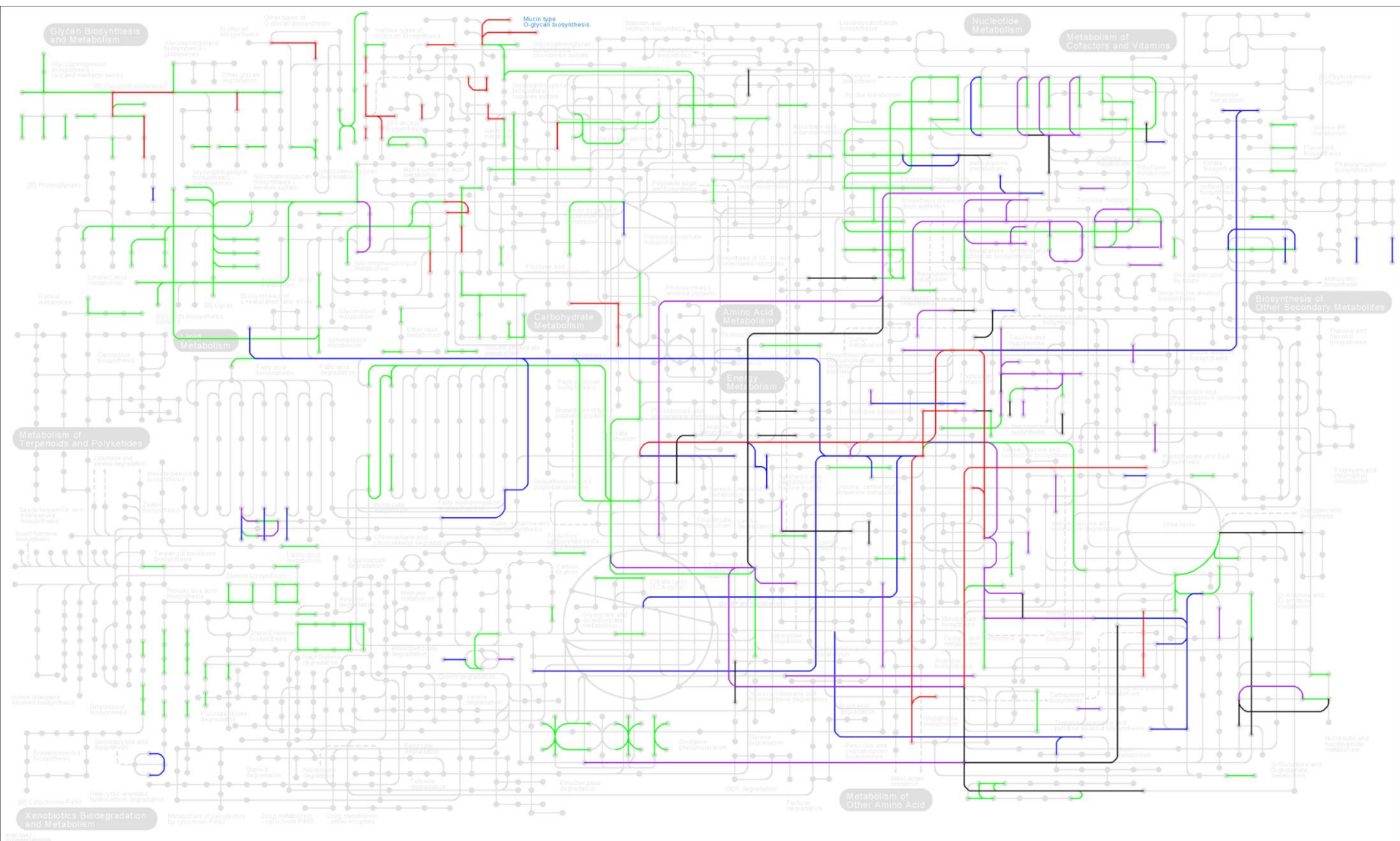

Supplementary Figure

Supplement: Supplementary Figure [file mmc2.pdf]
